# Impact of Organic Carbons Addition on the Enrichment Culture of Nitrifying Biofloc from Aquaculture Water: Process, Efficiency, and Microbial Community

**DOI:** 10.3390/microorganisms12040703

**Published:** 2024-03-30

**Authors:** Jiaqi Wu, Wujie Xu, Yu Xu, Haochang Su, Xiaojuan Hu, Yucheng Cao, Jianshe Zhang, Guoliang Wen

**Affiliations:** 1 National Engineering Research Center for Marine Aquaculture, Zhejiang Ocean University, Zhoushan 316022, China; wujiaqi3861@163.com (J.W.); zhangjianshe@zjou.edu.cn (J.Z.); 2 South China Sea Fisheries Research Institute, Chinese Academy of Fishery Sciences, Guangzhou 510300, China; xuyublq@163.com (Y.X.); su.haochang@163.com (H.S.); xinr129@163.com (X.H.); cyc_169@163.com (Y.C.); 3 Key Laboratory of South China Sea Fishery Resources Exploitation & Utilization, Ministry of Agriculture and Rural Affairs, Guangzhou 510300, China; 4 Southern Marine Science and Engineering Guangdong Laboratory (Zhuhai), Zhuhai 519082, China

**Keywords:** biofloc technology, organic carbon addition, enrichment process, nitrification efficiency, nitrifying bacteria, nitrifying gene

## Abstract

In this study, we developed a rapid and effective method for enriching the culture of nitrifying bioflocs (NBF) from aquacultural brackish water. The self-designed mixotrophic mediums with a single or mixed addition of sodium acetate, sodium citrate, and sucrose were used to investigate the enrichment process and nitrification efficiency of NBF in small-scale reactors. The results showed that NBF with an MLVSSs from 1170.4 mg L^−1^ to 2588.0 mg L^−1^ were successfully enriched in a period of less than 16 days. The citrate group performed the fastest enrichment time of 10 days, while the sucrose group had the highest biomass of 2588.0 ± 384.7 mg L^−1^. In situ testing showed that the highest nitrification efficiency was achieved in the citrate group, with an ammonia oxidation rate of 1.45 ± 0.34 mg N L^−1^ h^−1^, a net nitrification rate of 2.02 ± 0.20 mg N L^−1^ h^−1^, and a specific nitrification rate of 0.72 ± 0.14 mg N g^−1^ h^−1^. Metagenomic sequencing revealed that *Nitrosomonas* (0.0~1.0%) and *Nitrobacter* (10.1~26.5%) were dominant genera for AOB and NOB, respectively, both of which had the highest relative abundances in the citrate group. Linear regression analysis further demonstrated significantly positive linear relations between nitrification efficiencies and nitrifying bacterial genera and gene abundance in NBF. The results of this study provide an efficient enrichment culture method of NBF for the operation of biofloc technology aquaculture systems, which will further promote its wide application in modern intensive aquaculture.

## 1. Introduction

Aquaculture has become an important agricultural production activity, and it plays a key role in global food and nutrition security [1]. With the development of intensification, water nitrogen pollution and associated environmental problems during aquaculture are increasingly prominent [2]. Nitrogenous wastes, which are derived from the excretion of aquacultural animals and the microbial degradation of uneaten food, usually accumulate in intensive aquaculture systems. Among them, ammonia and nitrite are especially harmful to aquacultural animals [3]. Therefore, how to effectively remove these toxic nitrogenous compounds is a priority of water quality control for modern aquaculture.

As one of the most promising microbial technologies, biofloc technology (BFT) has gained great attention in nitrogenous waste removal for sustainable intensive aquaculture [4,5,6,7,8]. The basis of BFT is active bioflocs, which are suspended aggregates of various bacterial communities flocculated with organic particles in the body of aquaculture water [9,10,11]. The bacterial communities are the functional body of bioflocs, which are promoted and developed usually by supplementing organic carbons in the culture water for the harmful nitrogenous transformation and control in the aquaculture systems [4,11]. Two main pathways of nitrogen conversion are mediated by the bacterial communities of bioflocs, which are the autotrophic nitrification of ammonium-nitrogen (NH_4_^+^-N) to nitrite-nitrogen (NO_2_^−^-N) and further to nitrate-nitrogen (NO_3_^−^-N), and the heterotrophic assimilation of NH_4_^+^-N directly to bacterial biomass [7,11]. An increasing number of studies have shown that nitrification should be more substantial and beneficial for NH_4_^+^-N and NO_2_^−^-N removal than heterotrophic assimilation, as production intensity increases in the BFT aquaculture systems [12,13,14,15,16]. It is now widely recognized that the establishment of nitrification is the key process to reach the stability and maturity status of BFT aquaculture systems [7,14,15,17].

Over the past decade, BFT has been widely studied and applied in aquaculture for many shrimp and fish species [18,19]. In practice, significant rises of NH_4_^+^-N and NO_2_^−^-N and an uncertain duration of their peaks are often observed when applying BFT in the intensive aquaculture systems [14,15,17,20]. This is supposed to be related to the delayed establishment of nitrification function in the BFT systems, which is carried out by the attached nitrifying bacteria of bioflocs [7,14,20]. Previous studies have shown that inoculating mature nitrifying bioflocs was effective in the smooth operation of BFT aquaculture systems, as no obvious peaks of NH_4_^+^-N and NO_2_^−^-N were observed during the whole process [12,14,17,21,22,23]. Thereby, the pre-culture or enrichment of nitrifying bioflocs became an important and indispensable task before the initiation of the BFT aquaculture systems.

Nitrifying bacteria are generally chemoautotrophic bacteria, including ammonia-oxidizing bacteria (AOB) (e.g., *Nitrosomonas*) and nitrite-oxidizing bacteria (NOB) (e.g., *Nitrobacter*). They jointly execute a two-step process of nitrification, in which ammonia is first oxidized to nitrite by AOB and subsequently to nitrate by NOB. Due to slow growth and attachment characteristics, long periods are often required to obtain nitrifying bacterial consortia, especially for the enrichment from natural environment using autotrophic inorganic mediums [24,25,26]. Bioflocs are typically microbial aggregates, and their formation and development are accompanied by the attachment growth process of nitrifying bacteria [7,12,13]. To date, there have been reports on the enrichment of nitrifying bacterial consortia or heterotrophic bioflocs from aquatic systems [16,27,28], while no study has been conducted to explore the rapid and effective enrichment of nitrifying bioflocs.

In this study, we primarily aimed to establish a rapid and effective method for the enrichment culture of nitrifying bioflocs. Here, we hypothesized that an organic carbon addition can enhance the process and efficiency of the nitrifying biofloc enrichment culture from aquaculture water. To prove this, self-designed mixotrophic mediums with the addition of different organic carbon sources were used to investigate the enrichment process of nitrifying bioflocs. The nitrification efficiency of enriched nitrifying bioflocs was evaluated in terms of the in situ ammonia oxidation rate, net nitrification rate, and specific nitrification rate. Furthermore, the nitrifying bacterial community and functional genes of the enriched nitrifying bioflocs were profiled by metagenomic sequencing and were further correlated to nitrification efficiency.

## 2. Materials and Methods

### 2.1. Medium Preparation and Enrichment Experiment

The mixotrophic medium for the enrichment culture of nitrifying bioflocs contained the following (g L^−1^): powered shrimp feed (0.2), (NH_4_)_2_SO_4_ (0.1), NaNO_2_ (0.1), NaHCO_3_ (0.3), CaCl_2_ (0.5), MgSO_4_ (0.3), KH_2_PO_4_ (0.3), and 10 mL of trace element solution. The trace element solution contained the following (g L^−1^): FeSO_4_·7H_2_O (1.0), MnSO_4_·4H_2_O (1.0), ZnSO_4_ (0.1), CuSO_4_·5H_2_O (0.1), Na_2_MoO_4_·2H_2_O (0.1), and EDTA-Na_2_·2H_2_O (2.0). The powered shrimp feed had 40.0% protein, 8.0% lipid, 3.5% fiber, and 14% ash. The enrichment mediums were further prepared with mixotrophic medium by adding organic carbon compounds at a concentration of 0.3 g L^−1^. Sodium acetate (C_2_H_3_O_2_Na·3H_2_O), sodium citrate (C_6_H_5_O_7_Na_3_·2H_2_O), and sucrose (C_12_H_22_O_11_) were used as three kinds of organic carbon.

For the enrichment experiment, three groups were set up with a sodium acetate, sodium citrate, and sucrose addition of 0.3 g L^−1^, respectively; one group had a mixture addition of sodium acetate, sodium citrate, and sucrose at 0.1 g L^−1^ each, and the control group had no organic carbon addition. After adding organic carbons, the pH of the enrichment medium was adjusted to 7.8 with the addition of diluted hydrochloric acid (1 mol L^−1^).

### 2.2. Water Sample Collection for Seeding

Water samples were collected from an intensive brackish water pond at Guanlida Marine Bio Technology company’s shrimp farm in the city of Maoming, Guangdong province. The sampling pond is about 0.1 hectare, and it was stocked with 150 thousand post-larval of *Penaeus vanamei*. The shrimp were cultured for forty-six days under limited water exchange conditions. The basic characteristics of the culture water were as follows: salinity of 12 mg L^−1^, temperature of 29 °C, pH of 7.8, dissolved oxygen of 5.1 mg L^−1^, total alkalinity of 106 mg L^−1^ as CaCO_3_, NH_4_^+^-N of 3.2 mg L^−1^, NO_2_^−^-N of 3.5 mg L^−1^, and NO_3_^−^-N of 23.6 mg L^−1^. The pond water was collected with an organic glass hydrophore, and about eighty liters of the water was transported to the laboratory of the company.

### 2.3. Enrichment Reactor and Culture Process

The reactor was a cylindrical tank with a 16.0 cm inner diameter, 35 cm height, and 5.0 L effective volume. A discal air-stone was placed on the bottom of each reactor and connected to an air pump. Fifteen reactors were prepared and randomly assigned to the five experimental groups. Each experimental group had three reactors, and each reactor was filled with 4.5 L of respective enrichment mediums. Then, 0.5 L of the water sample was inoculated into each reactor, and the enrichment culture of nitrifying bioflocs started. The mixing and aeration of the reactors were achieved by bubbling the air of air-stones. The reactors were operated at room temperature (26~30 °C). The pH was kept at 7.6~8.0 by adding saturated sodium carbonate solution. The dissolved oxygen concentration was kept at 4.0~6.0 mg L^−1^. The experiment of enrichment culture continued until both NH_4_^+^-N and NO_2_^−^-N concentrations were stabilized below 1.0 mg L^−1^ in all reactors. Dechlorinated tap water was added to the reactors for the compensation of water loss due to evaporation and water sampling.

### 2.4. In Situ Nitrification Testing and Performance Analysis of Enriched Bioflocs

At the end of the enrichment culture, 0.5 g of (NH_4_)_2_SO_4_ and 0.5 g of NaNO_2_ were both added into each reactor to evaluate the nitrification efficiency of enriched nitrifying bioflocs. The calculated concentrations of added NH_4_^+^-N and NO_2_^−^-N were 21.2 mg L^−1^ and 20.3 mg L^−1^. The dynamic concentrations of NH_4_^+^-N, NO_2_^−^-N, NO_3_^−^-N, and total N (TIN) were detected and calculated for each reactor. The in situ ammonia oxidation rate (AOR), net nitrification rate (NNR), and specific nitrification rate (SNR) were calculated using the following equations:

AOR (mg N L^−1^ h^−1^) = [C_0_(NH_4_^+^-N) − C_t_(NH_4_^+^-N)]/∆t; (1)

NNR (mg N L^−1^ h^−1^) = [C_t_(NO_3_^−^-N) − C_0_(NO_3_^−^-N)]/∆t; (2)

SNR (mg N g^−1^ h^−1^) = [C_t_(NO_3_^−^-N) − C_0_(NO_3_^−^-N)]/MLVSS/∆t; (3)

where C_0_(NH_4_^+^-N) and C_t_(NH_4_^+^-N) are the concentrations of NH_4_^+^-N detected initially and one day later, C_0_(NO_3_^−^-N) and C_t_(NO_3_^−^-N) are the concentrations of NO_3_^−^-N detected initially and one day later, MLVSS is the mixed liquor volatile suspended solids and represents the biomass concentration of bioflocs, and ∆t is the reaction time of 24 h for one day.

### 2.5. Water Bioflocs and Various Nitrogen Detection Analysis

Water samples were collected by 50 mL plastic bottles from each reactor for biofloc biomass and various from nitrogen concentration analysis; the sampling was conducted every two days in the enrichment experiment and daily in the nitrification testing. Total mixed liquor volatile suspended solids (MLVSSs), NH_4_^+^-N, NO_2_^−^-N, NO_3_^−^-N, and total N (TN), were detected following the “Standard methods for the examination of water and wastewater” [29]. TIN was calculated as the sum of NH_4_^+^-N, NO_2_^−^-N, and NO_3_^−^-N. At the end of the enrichment experiment, actual TN was detected for each reactor; total organic N (TON) was calculated by subtracting TIN from actual TN; and N loss was calculated by subtracting actual TN from input TN. Input TN represented the total N of the input in the reactor, including the N content of both the initial inoculated water sample and the enrichment medium.

### 2.6. Biofloc DNA Extraction, Metagenomic Sequencing, and Bioinformatics Analysis

At the end of the enrichment experiment, 50 mL of mixed water was also collected by sterile bottles from each reactor and then filtered through a 0.2 μm pore-size polycarbonate membrane filter (Millipore, Bedford, MA, USA) to obtain the biofloc sample. Total genomic DNA was extracted from biofloc samples using the E.Z.N.A.^®^ Soil DNA Kit (Omega Bio-Tek, Norcross, GA, USA), and after the measurement of quantity and quality, the extracted DNA was then sent for metagenomic sequencing at Wekemo Tech Group Co., Ltd., Shenzhen, China. Individual libraries were constructed using the NEBNext@ UltraTM DNA Library Prep Kit (NEB, Ipswich, MA, USA), and DNA sequencing was performed on the Illumina NovaSeq platform (Illumina, San Diego, CA, USA) using a 2 × 150 bp paired-end read protocol. The raw sequence data generated in this study were deposited into the NCBI Short Read Archive database (accession number: PRJNA1071834).

The raw sequences were preprocessed using Kneaddata (v0.10.0, https://github.com/biobakery/kneaddata (accessed on 22 March 2023)) for quality control. Then, all clean sequences were annotated and classified using kraken2 (v2.0.8-beta, http://ccb.jhu.edu/software/kraken2/ (accessed on 22 March 2023)) and a self-built microbial database (sequences were screened from NT nucleic acid database and RefSeq whole genome database of NCBI) to characterize the taxonomic composition of the metagenomic dataset of the samples. Bracken (v2.5.0, https://ccb.jhu.edu/software/bracken/index.shtml (accessed on 10 April 2023)) was used to estimate the species-level abundance of metagenomic samples.

The clean sequences were also assembled into contigs using MEGAHIT v1.1.2 [30], and gene sequences in all contigs were predicted using Prodigal v2.6.3 [31]. Then the de-redundant gene was obtained using Cd-hit v4.6.1 [32], quantified using Salmon v0.14.1 [33], and translated into protein sequences for subsequent blast and functional annotation against Kyoto Encyclopedia of Genes and Genomes (KEGG v94.2, http://www.genome.jp/kegg/ (accessed on 12 April 2023)) using Eggnog-mapper v2.0.1 based on DIAMOND [34,35]. The targeted N-cycling genes were filtered out from the metagenomic samples. The abundance of the de-redundant gene was annotated to the same gene family and is presented as transcripts per kilobase million (TPM).

### 2.7. Statistical Analysis

All statistical analyses were performed using IBM SPSS Statistics 20.0 software for Windows (IBM Corporation, Armonk, NY, USA). The normality of data was evaluated using the Shapiro–Wilk test. The data means were analyzed using one way ANOVA after conducting Levene’s test of homogeneity of variance. Difference was considered significant at *p* < 0.05. The percentage data were transformed with arcsine square root before analysis.

## 3. Results and Discussion

Nitrogen wastes, especially harmful ammonia and nitrite, are major stressors of water environments in intensive aquaculture systems. BFT provides a microbial ecology pathway to solve this problem, and its application basis is the nitrification of bioflocs in the in situ water of aquaculture systems. In this study, we established a rapid and effective method for the enrichment culture of nitrifying bioflocs in small-scale reactors. The addition of different organic carbons in the enrichment medium altered the nitrification process and efficiency of the enriched bioflocs, which should be the result of differences in the abundance of *Nitrobacter* and *Nitrosomonas* and related nitrifying genes.

### 3.1. Enrichment Process of Nitrifying Bioflocs and The Effects of Organic Carbons Addition

**Figure 1:**
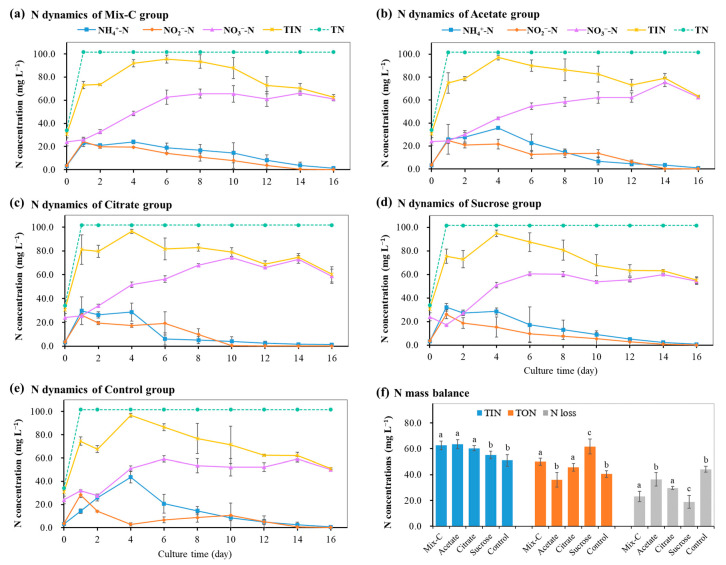
N dynamics and N mass balance during the enrichment process of nitrifying bioflocs in the reactors by different organic carbon additions (means ± S.D., n = 3). Mix-C: mixture of sodium acetate, sodium citrate, and sucrose; Control: no organic carbon addition; TIN: total inorganic nitrogen; TN: total nitrogen of input. Different letters in (**f**) indicate significant differences among five groups (*p* < 0.05).

**Figure 2:**
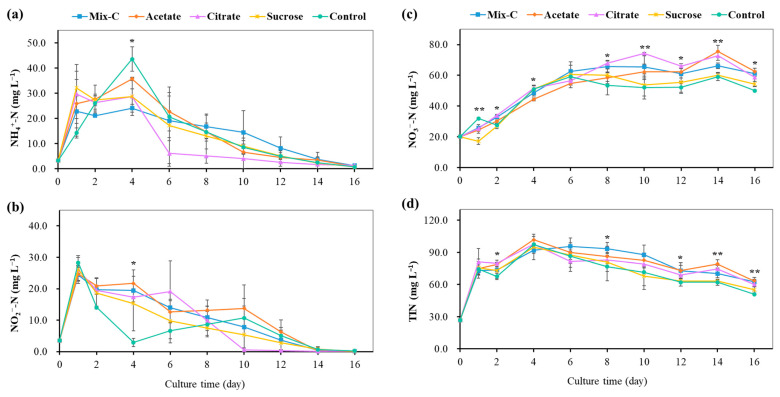
The effect of different organic carbon additions on the enrichment process of nitrifying bioflocs in the reactors (means ± S.D., n = 3). (**a**): Concentration change of NH_4_^+^-N, (**b**): Concentration change of NO_2_^−^-N, (**c**): Concentration change of NO_3_^−^-N, (**d**): Concentration change of TIN. Mix-C: mixture of sodium acetate, sodium citrate and sucrose; Control: no organic carbon addition; TIN: total inorganic nitrogen. The asterisk indicates significant differences among five groups (*, *p* < 0.05; **, *p* < 0.01).

In aquaculture practice, artificial feeds and other high-carbon matters are usually used to culture bioflocs, and the establishment of nitrification was recognized as the sign of mature bioflocs [7,12,14,20,22]. In this study, a mixotrophic medium was designed and used, which was modified from the combination of autotrophic medium and artificial feed. Autotrophic medium had both NH_4_^+^-N and NO_2_^−^-N substrates for the concomitant growth of ammonia- and nitrite-oxidizing bacteria [36,37]. Artificial feed provided organic nitrogen and carbon sources for bacterial growth and, meanwhile, simulated the feeding environment of aquaculture systems. Moreover, three kinds of low molecular and commonly used organic carbon sources, including sodium acetate, sodium citrate, and sucrose, were added into the enrichment medium for the enhancement of the enrichment process [27,38]. Good results were achieved in the enrichment period and biomass production compared to previous similar studies [16,24,25,37]. The enrichment process of nitrifying bioflocs was clearly shown by the concentration changes of NH_4_^+^-N, NO_2_^−^-N, NO_3_^−^-N, TIN, and input TN in the reactors with different organic carbon additions (Figure 1). From day one of the enrichment process, the NH_4_^+^-N concentration firstly increased and then decreased; the NO_2_^−^-N concentration declined gradually; and the NO_3_^−^-N concentration increased rapidly and then fluctuated slightly during a 16-day culture period (Figure 1 and Figure 2), indicating the establishment of the nitrification process along with the enrichment growth of nitrifying bioflocs in reactors. Organic nitrogen in the feed could be decomposed and ammoniated into NH_4_^+^-N by heterotrophic bacteria in the initial stage [16], which accounted for the observed increase of NH_4_^+^-N during the first four days (Figure 2a).

Although there were similar enrichment processes in all reactors, the N mass balance showed significant differences among the five groups with different organic carbons addition (Figure 1f). It was found that the citrate group had the highest concentration of TIN, the sucrose group had the highest concentration of TON, and the control group had the highest concentration of N loss in the reactors (Figure 1f). This indicated that, while NH_4_^+^-N and NO_2_^−^-N were used for the nitrification and growth of nitrifying bacteria, there also existed N loss induced by denitrification during the enrichment of the bioflocs [39]. This could be supported by the abundant denitrifying bacteria and functional genes in the enriched bioflocs by metagenomic analysis below hereinafter. Further, comparison analysis also showed that different organic carbon additions had significant effects on the concentration dynamics of NH_4_^+^-N, NO_2_^−^-N, NO_3_^−^-N, and TIN during the enrichment process of nitrifying bioflocs (Figure 2). For the establishment of time of complete nitrification, the citrate group showed the fastest enrichment of nitrifying bioflocs, and it only took ten days (Figure 2).

**Figure 3:**
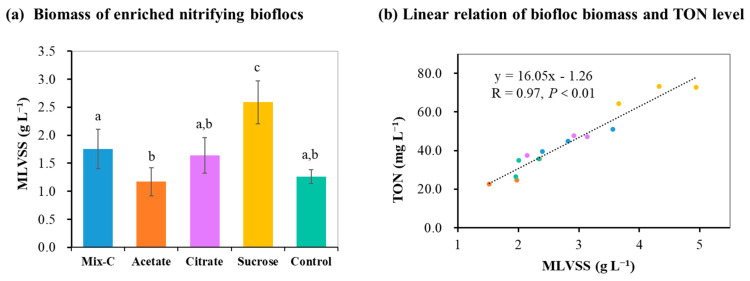
Effects of different organic carbon additions on the biomass production of nitrifying bioflocs in the reactors (means ± S.D., n = 3). Mix-C: mixture of sodium acetate, sodium citrate, and sucrose; Control: no organic carbon addition; MLVSS: mixed liquor volatile suspended solid; TON: total organic nitrogen. Different letters indicate significant differences among the five groups (*p* < 0.05). The dots with five colors represent five groups.

The addition of specific organic carbon could not only contribute to shortening the enrichment period but also to increasing biomass production during the culture of nitrifying bioflocs. It was shown that the nitrification process was completely established in all reactors on day 16, and the biomass production of nitrifying bioflocs in terms of MLVSS was between 1170.4 mg L^−1^ and 2588.0 mg L^−1^ (Figure 3a). Significant differences in MLVSS were observed among five groups, with the sucrose group the highest and the control group lowest. This indicates that the promotional effects of the three organic carbon sources in addition to the production of bioflocs had varying extents. The organic carbon addition could promote the growth of heterotrophic nitrifying bacteria to form bioflocs, which further provides an attachment matrix for autotrophic nitrifying bacteria growth [12,39,40]. Moreover, a significantly positive linear relation (R = 0.97, *p* < 0.01) of biofloc biomass and TON level could be found (Figure 3b), indicating that TON in the reactor should be mainly derived from cultured bioflocs [41]. For biomass production, the sucrose group showed the highest enriched biomass of nitrifying bioflocs, with MLVSSs of 2588.0 ± 384.7 mg L^−1^ (Figure 3a).

### 3.2. Nitrifying Bacteria and Genes of Enriched Bioflocs, and the Effects of Organic Carbons Addition

**Figure 4:**
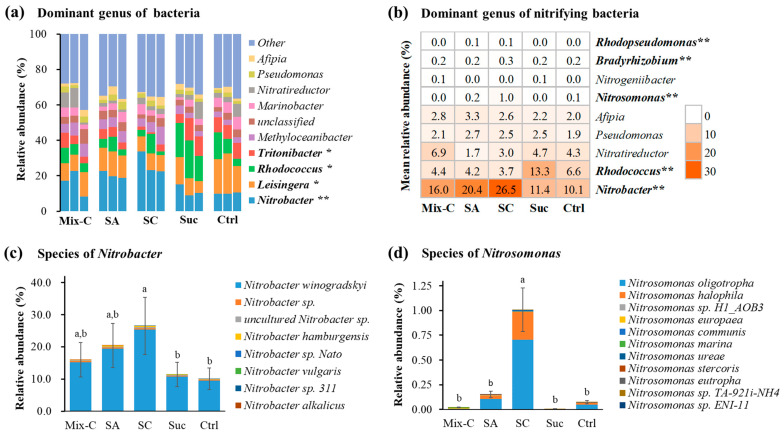
Effects of different organic carbon additions on nitrifying bacteria of enriched bioflocs identified from metagenomic sequencing (means ± S.D., n = 3). Mix-C: mixture of sodium acetate, sodium citrate, and sucrose; SA: sodium acetate; SC: sodium citrate; Suc: sucrose; Control: no organic carbon addition. The asterisk indicates significant differences among the five groups (*, *p* < 0.05; **, *p* < 0.01); different letters indicate significant differences among the five groups (*p* < 0.05).

Metagenomic sequencing revealed that both ammonia- and nitrite-oxidizing bacteria were simultaneously enriched in the nitrifying bioflocs. *Nitrobacter*, *Leisingera*, *Rhodococcus*, and *Tritonibacter* were the top four dominant bacterial genera shared by all reactors, and they showed significant differences in the relative abundance among five groups (Figure 4a). For nitrifying bacteria, *Nitrobacter*, *Rhodococcus*, *Nitratireductor*, *Pseudomonas*, *Afipia*, *Nitrosomonas*, *Nitrogeniibacter*, *Bradyrhizobium*, and *Rhodopseudomonas* were found to be dominant genera; among them, *Nitrobacter*, *Rhodococcus*, *Nitrosomonas*, *Bradyrhizobium*, and *Rhodopseudomonas* showed significant differences in the relative abundance among five groups (Figure 4b). *Nitrosomonas* was the most abundant genus of AOB and accounted for 0.0~1.0%, while *Nitrobacter* was the most abundant genus of NOB and accounted for 10.1~26.5% (Figure 4b). For both *Nitrosomonas* and *Nitrobacter*, the highest relative abundances were detected in the citrate group (Figure 4b). Eight species belonging to *Nitrobacter* could be detected, and *Nitrobacter winogradskyi* was the most dominant species, while eleven species belonging to *Nitrosomonas* could be detected, and *Nitrosomonas oligotropha* was the most dominant species (Figure 4c).

In this study, both *Nitrosomonas* (e.g., *N. oligotropha*) and *Nitrobacter* (e.g., *N. winogradskyi*) were detected as dominant nitrifying bacteria in the enriched bioflocs, which are known to be major players of ammonia oxidation and nitrite oxidation respectively [42,43]. The two functionally synergistic populations are commonly found in aquatic environments or aquaculture systems [11,42,44,45] and are known to be widely involved in the two-step process of nitrification. The *Nitrosomonas* and *Nitrobacter* species are usually assumed to be r-strategists AOB and NOB, respectively, both of which can grow quickly at high substrate concentrations [27,46]. The high abundances of *Nitrosomonas* and *Nitrobacter* enriched in the nitrifying bioflocs illustrate that the designed and used mixotrophic medium of this study provided an appropriate and high-nutrient environment for the enrichment culture of the two r-strategist nitrifying populations in the reactors [47]. Moreover, these two nitrifying populations could have the ability to attach and form bioflocs [13,15]. It should be noted that the abundance of *Nitrobacter* (10.1~26.5%) was far higher than *Nitrosomonas* (0.0~1.0%) in the enriched bioflocs. This could guarantee that complete nitrification was achieved without the accumulation of nitrite.

**Figure 5:**
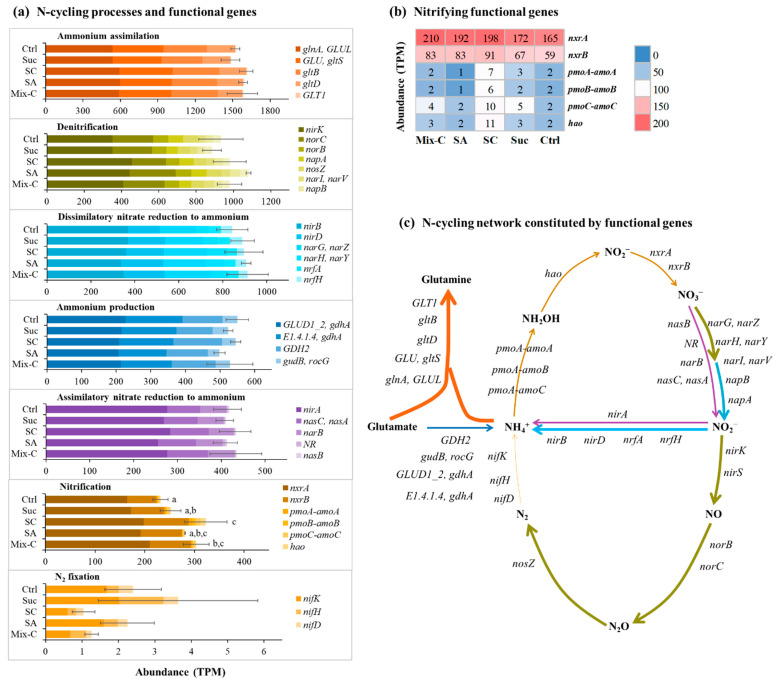
Effects of different organic carbon additions on the N-cycling functional genes of enriched bioflocs by analyzing metagenomic data against the KEGG pathway database (means ± S.D., n = 3). For N-cycling processes and involved functional genes, different colors of arrow indicate specific N-transformation processes, and the thickness of each arrow is proportional to the total abundance of involved functional genes. Mix-C: mixture of sodium acetate, sodium citrate, and sucrose; SA: sodium acetate; SC: sodium citrate; Suc: sucrose; Ctrl: control, no organic carbon addition. Different letters indicate significant differences among the five groups (*p* < 0.05).

Meanwhile, a subset of 36 functional genes was identified from the enriched bioflocs, which comprised seven N-transformation processes: nitrification, denitrification, assimilatory nitrate reduction to ammonium, dissimilatory nitrate reduction to ammonium, ammonium assimilation, ammonium production, and N_2_ fixation (Figure 5). Among the five groups, there were no significant differences in the abundance of gene families for these N-transformation processes, except for nitrification, and ammonium assimilation had the highest abundance of gene families compared to the other N-transformation processes (Figure 5a). Importantly, the abundance of gene families for the nitrification process showed significant differences among the five groups with different organic carbon additions, and the highest could be found in the citrate group (Figure 5a,b). This difference in nitrifying gene abundance could be found to be in accordance with the difference of the *Nitrobacter* abundance (Figure 4c and Figure 5a), indicating the uniformity of the structure and function of the enriched nitrifying bioflocs. Five nitrifying functional genes of *nxrA*, *nxrB*, *pmoA-amoA*, *pmoB-amoB*, *pmoC-amoC*, and *hao* could be found in the enriched bioflocs, all of which showed differences among the five groups (Figure 5b). The *nxrA* gene showed the highest abundance in nitrifying functional genes, which encoded the alpha subunit of nitrite oxidoreductase catalyzing the oxidation of nitrite to nitrate [48]. It is very meaningful to find out that these functional genes constituted a complete and complex N-cycling network in the enriched bioflocs (Figure 5c) [49].

### 3.3. Nitrification Efficiency of Enriched Bioflocs, and Their Relations with Nitrifying Bacteria and Genes

**Figure 6:**
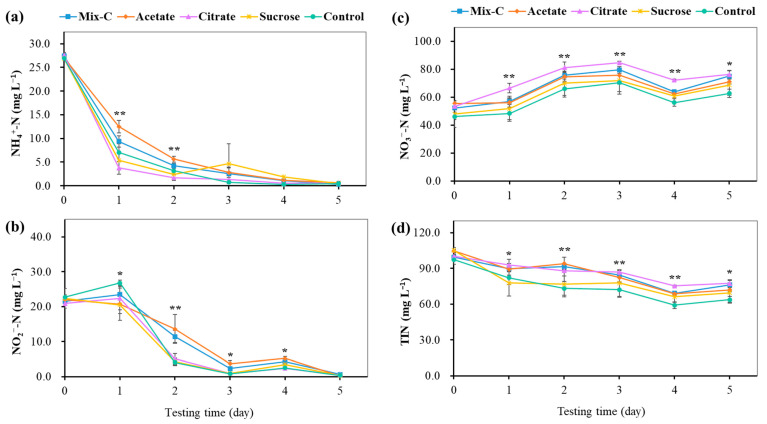
Nitrification performance of nitrifying bioflocs enriched from different organic carbon additions in the reactors. (**a**): Concentration change of NH_4_^+^-N, (**b**): Concentration change of NO_2_^−^-N, (**c**): Concentration change of NO_3_^−^-N, (**d**): Concentration change of TIN. Mix-C: mixture of sodium acetate, sodium citrate, and sucrose; Control: no organic carbon addition; TIN: total inorganic nitrogen. The asterisk indicates significant differences among the five groups (*, *p* < 0.05; **, *p* < 0.01).

After the enrichment culture of nitrifying bioflocs, the nitrification efficiency was further evaluated through an in situ test spiked with high concentrations of NH_4_^+^-N and NO_2_^−^-N in this study. During the five days of testing, a rapid decrease in NH_4_^+^-N concentration, a slight increase, followed by a sharp decline of NO_2_^−^-N concentration, a gradual increase followed by a slight fluctuation of NO_3_^−^-N concentration, and a slow decrease of TIN concentration were observed (Figure 6). The concentration dynamics of NH_4_^+^-N, NO_2_^−^-N, NO_3_^−^-N, and TIN clearly demonstrated the efficient nitrifying activity of enriched bioflocs in the reactors, which were comparable to relative studies [28,42,45]. It is worthy to note that the nitrifying bioflocs enriched from different organic carbon additions showed some differences in nitrification performance (Figure 6). From the view of the rapid nitrification of NH_4_^+^-N and NO_2_^−^-N to NO_3_^−^-N, the citrate group showed the best performance. The further comparison of nitrification efficiencies among the five groups evidenced this. The results showed that the highest nitrification efficiencies were achieved in the citrate group, with an ammonia oxidation rate of 1.45 ± 0.34 mg N L^−1^ h^−1^, net nitrification rate of 2.02 ± 0.20 mg N L^−1^ h^−1^, and specific nitrification rate of 0.72 ± 0.14 mg N g^−1^ h^−1^ (Table 1).

**Figure 7:**
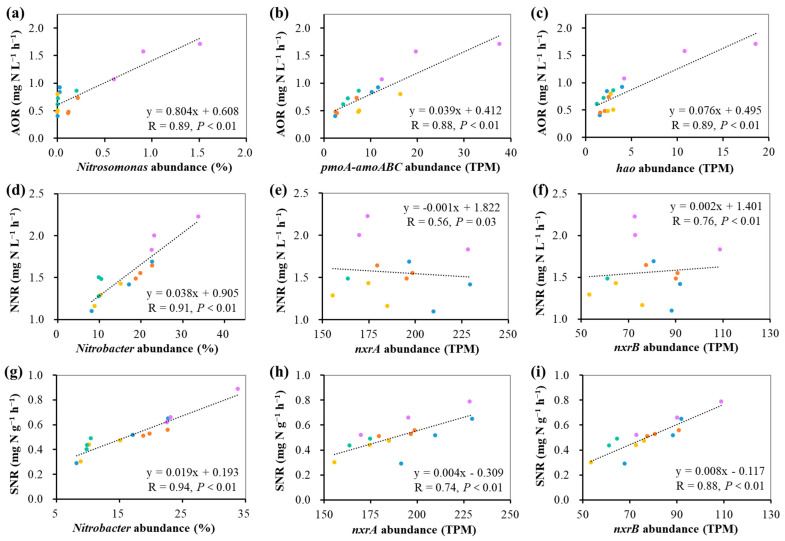
Relationships of nitrification efficiencies and nitrifying bacteria and genes of nitrifying bioflocs enriched from different organic carbon additions in the reactors based on linear regression analysis (n = 15). The dots with five colors represent five groups. (**a**): Linear relation of AOR and *Nitrosomonas* abundance, (**b**): Linear relation of AOR and *pmoA-amoABC* abundance, (**c**): Linear relation of AOR and *hao* abundance, (**d**): Linear relation of NNR and *Nitrobacter* abundance, (**e**): Linear relation of NNR and *nxrA* abundance, (**f**): Linear relation of NNR and *nxrB* abundance, (**g**): Linear relation of SNR and *Nitrobacter* abundance, (**h**): Linear relation of SNR and *nxrA* abundance, (**i**): Linear relation of SNR and *nxrB* abundance. AOR: ammonia oxidation rate; NNR: net nitrification rate; SNR: specific nitrification rate.

Nitrifying bacteria are the executors of the nitrification process of enriched bioflocs, and the nitrification activity should be correlated to the abundance of nitrifying bacteria. Significantly positive linear relations were found between nitrification efficiencies and functionally synergistic populations and genes of nitrifying bacteria in enriched bioflocs from the fifteen reactors (Figure 7). The ammonia oxidation rate was significantly positively correlated with the abundance of the *Nitrosomonas* genus (R = 0.89, *p* < 0.01), *pmoA-amoABC* gene (R = 0.88, *p* < 0.01), and *hao* gene (R = 0.88, *p* < 0.01) (Figure 7a–c). This further confirmed that *Nitrosomonas* could play a leading role in ammonia oxidation, and *pmoA-amoABC* and *hao* were the main involved functional genes. The net nitrification rate was significantly positively correlated with the abundance of *Nitrobacter* genus (R = 0.78, *p* < 0.01), *nxrA* gene (R = 0.76, *p* < 0.01), and *nxrB* gene (R = 0.74, *p* < 0.01) (Figure 7d–f). The specific nitrification rate was significantly positively correlated with the abundance of the *Nitrobacter* genus (R = 0.94, *p* < 0.01), *nxrA* gene (R = 0.74, *p* < 0.01), and *nxrB* gene (R = 0.88, *p* < 0.01) (Figure 7g–i). These results further confirmed that *Nitrobacter* could play a predominant role in the two-step nitrification process, and *nxrA* and *nxrB* were the main functional genes involved in nitrite oxidation. The above results closely established links of nitrifying functional genes, nitrifying bacterial populations, and nitrification efficiency, which demonstrated the consistency of structure and function of enriched nitrifying bioflocs.

## 4. Conclusions

This study established a rapid and effective method for the enrichment culture of nitrifying bioflocs from aquacultural brackish water. The results showed that the addition of sodium acetate, sodium citrate, and sucrose had different enhancement impacts on the enrichment process, nitrification efficiency, and bacterial community of nitrifying bioflocs. Nitrifying bioflocs could be successfully enriched with up to 2588.0 mg L^−1^ MLVSS in less than 16 days, and the citrate group performed the best in the enrichment process, nitrification efficiency, and nitrifying bacterial genera and gene abundance. *Nitrosomonas* (0.0~1.0%) and *Nitrobacter* (10.1~26.5%) were dominant genera for AOB and NOB, respectively, in the enriched nitrifying bioflocs, both of which significantly positively correlated to the involved functional genes and nitrification efficiencies. Further research is needed to evaluate practical application effects of the enriched nitrifying bioflocs obtained in this study.

## Figures and Tables

**Table 1:** Nitrification efficiencies of nitrifying bioflocs enriched from different organic carbon additions in the reactors. Mix-C: mixture of sodium acetate, sodium citrate, and sucrose; Control: no organic carbon addition.

Nitrification Efficiency	Mix-C	Acetate	Citrate	Sucrose	Control
Ammonia oxidation rate (mg N L^−1^ h^−1^)	0.72 ± 0.28 ^a^	0.55 ± 0.16 ^a^	1.45 ± 0.34 ^b^	0.59 ± 0.18 ^a^	0.73 ± 0.12 ^a^
Net nitrification rate (mg N L^−1^ h^−1^)	1.40 ± 0.30 ^a^	1.56 ± 0.08 ^a^	2.02 ± 0.20 ^b^	1.29 ± 0.13 ^a^	1.42 ± 0.13 ^a^
Specific nitrification rate (mg N g^−1^ h^−1^)	0.49 ± 0.18 ^a^	0.53 ± 0.02 ^a,b^	0.72 ± 0.14 ^b^	0.40 ± 0.09 ^a^	0.44 ± 0.05 ^a^

Different letters indicate significant differences among the five groups (*p* < 0.05).

## Data Availability

The raw read datasets for this research can be found in the NCBI Sequence Read Archive database (Accession No. PRJNA1071834) (https://www.ncbi.nlm.nih.gov/sra/).
